# Fentanyl, carfentanil and other fentanyl analogues in Canada’s illicit opioid supply: A cross-sectional study

**DOI:** 10.1016/j.dadr.2024.100240

**Published:** 2024-05-23

**Authors:** Robert A. Kleinman

**Affiliations:** a Centre for Addiction and Mental Health, Toronto, ON, Canada; b Department of Psychiatry, University of Toronto, Toronto, ON, Canada

**Keywords:** Opioids, Fentanyl, Drug supply, Carfentanil, Fentanyl analogues

## Abstract

**Background:**

Despite the increase in fentanyl-involved overdose deaths in Canada, there have been no national-level studies evaluating the proportion of illicit opioids containing fentanyl or fentanyl analogues in Canada.

**Methods:**

This cross-sectional exploratory study characterized trends in fentanyl, carfentanil and other fentanyl analogues within opioids seized by law enforcement agencies in Canada from 2012 to 2022 and submitted to the Health Canada Drug Analysis Service (DAS). Analyses were stratified by province/region. Mann-Kandell tests were used to test for trends.

**Results:**

A total of 157,616 samples containing any opioid (“opioid-containing samples”) were submitted to the DAS from Canadian provinces between 2012 and 2022, of which 81,165 (51.5%) contained fentanyl or a fentanyl analogue. The percentage of opioid-containing samples that were positive for fentanyl or a fentanyl analogue increased from 3.0% (95% CI: 2.6–3.4%) in 2012–68.3% (67.7–68.9%) in 2022 (p < 0.001 for trend). The percentage of opioid-containing samples that were positive for fentanyl or a fentanyl analogue increased between 2012 and 2022 in all regions. In 2022, the percentage of samples containing fentanyl or an analogue followed an east-to-west gradient: 15.8% (13.3–18.6%) of samples in Atlantic Canada and 84.7% (83.6–85.7%) in British Columbia. Carfentanil was present in 4.9% (4.6–5.2%) of opioid-containing samples in Canada in 2022 and 19.7% (18.3–21.2%) of opioid-containing samples in Alberta.

**Conclusions:**

The illicit opioid supply in Canada increasingly contains toxic synthetic opioids. As of 2022, important regional differences existed in the illicit opioid supply in Canada.

## Highlights

• In 2012, 3% of opioid samples submitted for analysis contained fentanyl or analogs.
• In 2022, 68% of opioid samples submitted for analysis contained fentanyl or analogs.
• Fentanyl positivity was highest in British Columbia and lowest in Atlantic Canada.
• Carfentanil was present in almost 20% of opioid-containing samples in Alberta in 2022.

## 1 Introduction

Since 2015, there has been a substantial increase in the involvement of synthetic opioids, most commonly fentanyl and fentanyl analogues, in opioid-involved overdose deaths in Canada and the United States (Public Health Agency of Canada, 2023, Spencer et al., 2022). Local studies have characterized the presence of fentanyl in samples submitted to drug checking services, and national surveillance reports have indicated that the number of drug seizures by law enforcement containing fentanyl has increased (Government of Canada, 2023, Kennedy et al., 2023, Toronto’s Drug Checking Service Working Group, 2022, Tobias et al., 2022). To deepen the understanding of the presence of synthetic opioids within the illicit opioid supply across Canada, this study characterizes trends in the proportions of seized opioids containing fentanyl or its analogues across Canada.

For individuals who use drugs and clinicians treating patients with opioid use disorder, understanding the region-specific proportions of illicit opioid samples containing fentanyl has important potential implications for the risks associated with illicit opioid use (Kennedy et al., 2023, Zibbell et al., 2023), treatment of opioid withdrawal ((Thakrar and Kleinman, 2022), initiation of medications for opioid use disorder (Bromley et al., 2021, Weimer et al., 2023), and inpatient care for patients with OUD (Kleinman and Thakrar, 2023, Kleinman and Wakeman, 2022). For policymakers, understanding the proportion of the illicit opioid supply that contains fentanyl provides important information to tailor systems of care to needs of individuals with OUD.

## 2 Methods

### 2.1 Data sources

Data were obtained from the Health Canada Drug Analysis Service (DAS) on the number of fentanyl-containing and opioid-containing drug samples submitted between 2012 and 2022. Data were obtained from publicly released data and special data requests (Health Canada, 2023). Drug samples from illicit drug seizures are voluntarily submitted by law enforcement agencies to the DAS for analysis. The DAS describes their analytic methods as follows: “[The] DAS uses advanced and standardized analytical methods to analyze exhibits that are submitted to its laboratories. All Health Canada DAS laboratories are accredited as per the International Organization for Standardization (ISO 17025) and every analytical method used by Health Canada is recognized internationally as meeting or exceeding technical requirements for drug testing. Multiple types of tests and equipment are used to carry out our analysis and confirm the presence of controlled substances, such as Gas Chromatography-Mass Spectrometry (GC-MS), Liquid Chromatography-Mass Spectrometry (LC-MS) and Infrared Spectrometry (IR).” (Drug Analysis Service, personal communication, 2023)

### 2.2 Data Analysis

In this cross-sectional exploratory study, the proportions of opioid-containing samples that contained fentanyl, carfentanil or other fentanyl analogues were determined for each year. Analyses were stratified based on the province / region of origin of the sample. Results from the Atlantic provinces (Newfoundland and Labrador, Price Edward Island, Nova Scotia, and New Brunswick) were pooled in the analyses given the smaller populations of these provinces. All opioid samples submitted to the DAS were included in the analysis. Data from the territories were excluded from all analyses due to the low number of opioid-containing samples from the territories (n = 161 over 11 years).

Confidence intervals were calculated with the Clopper-Pearson method, assuming a binomial distribution for proportions. Mann-Kendall tests were used to evaluate for trends nationally and within each region. A Holm-Bonferroni correction was used to account for multiple testing (n = 8) for the main analysis of fentanyl- and fentanyl-analogue positivity.

To further describe the illicit opioid supply within each region, trends in fentanyl, carfentanil and other analogue positivity were further delineated. The proportion of each compound was calculated within both a) opioid-containing samples and b) samples containing any of fentanyl or fentanyl analogues. Confidence intervals for these proportions were calculated with the Clopper-Pearson method assuming a binomial distribution. Data was analyzed using R 4.2.3 (Foundation for Statistical Computing) between February 28, 2023 and September 18, 2023.

### 2.3 Ethics approval and reporting standards

This study was determined to be exempt from research ethics board approval by the Centre for Addiction and Mental Health Research Ethics Board. The DAS provided permission for publication of and analyses with data generated through special requests with the inclusion of the disclaimer found in the disclaimer section (Drug Analysis Service, personal communication, 2023). The study follows the Strengthening the Reporting of Observational Studies in Epidemiology (STROBE) guidelines (Elm et al., 2007).

## 3 Results

A total of 157,616 opioid-containing drug samples were submitted to the DAS from Canadian provinces between 2012 and 2022, of which 81,165 (51.5%) contained fentanyl or a fentanyl analogue. The percentage of opioid-containing samples that were positive for fentanyl or a fentanyl analogue increased from 3.0% (95% CI: 2.6–3.4%) in 2012–68.3% (67.7–68.9%) in 2022 (p<0.001 for trend). Of the 81,165 samples that contained fentanyl or a fentanyl analogue, 76,711 (94.5% [94.4–94.7%]) contained fentanyl, 5,830 (7.2% [7.0–7.4%]) contained carfentanil, and 5744 (7.1%) contained another fentanyl analogue. In 2022, 15,607 samples contained fentanyl or an analogue, of which 15,230 (97.6%[97.3–97.8%]) contained fentanyl, 1,116 (7.2%[6.8–7.6%]) contained carfentanil, and 707 (4.5%[4.2–4.9%]) contained another fentanyl analogue.

### 3.1 Provincial and regional trends

**Fig. 1:**
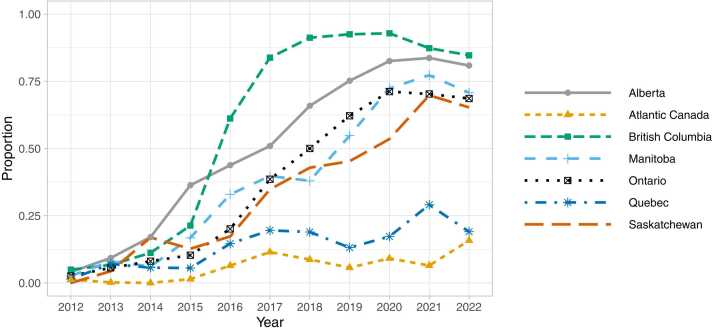
Proportion of opioid-containing samples with fentanyl or a fentanyl analogue, by province/region, 2012–2022.

In British Columbia, rates of fentanyl or fentanyl analogue positivity in opioid containing samples increased from 4.9% (3.9% - 6.1%) in 2012 to 21.3% (19.9%-22.9%) in 2015. Percentages then increased rapidly from 2016 (61.2% [59.4–62.9%]) through 2018 (91.2% [90.4–92.0%]) before decreasing through 2022 (84.7% [83.6–85.7%]). In Alberta, rates increased from 2012 (4.1% [2.4–6.3%] through 2015 (36.4% [34.0–38.8%]), had minimal change from 2015 through 2017 and increased in 2018 (54.9%[52.8–57.0]) through 2022 (80.9% [79.4–82.3%]). In Ontario, rates of fentanyl or fentanyl analogue positivity increased gradually from 2.6% (2.1–3.1%) in 2012–20.1% (18.9–21.3%) in 2016, with increases from 2017 (38.6% [37.3–39.8%]) through 2022 (68.6% [67.8–69.4%]. Trends in Saskatchewan and Manitoba were similar to those in Ontario (Fig. 1, Table 1).

**Table 1:** Percentage of illicit opioid-containing samples with fentanyl or fentanyl analogues, by province/region and year.

	**2012**	**2013**	**2014**	**2015**	**2016**	**2017**	**2018**	**2019**	**2020**	**2021**	**2022**	**p-value**
Total	3 (2.6–3.4)	6 (5.5–6.5)	9.4 (8.8–10)	16.3 (15.6–17)	34.3 (33.5–35.2)	51 (50.2–51.8)	60 (59.2–60.8)	66.9 (66.3–67.6)	74.4 (73.7–75)	72.5 (72–73.1)	68.3 (67.7–68.9)	<0.001
British Columbia	4.9 (3.9–6.1)	6.7 (5.6–7.9)	11.2 (9.9–12.6)	21.3 (19.9–22.9)	61.2 (59.4–62.9)	83.8 (82.7–84.9)	91.2 (90.4–92)	92.5 (91.7–93.2)	92.9 (92.1–93.5)	87.3 (86.4–88.2)	84.7 (83.6–85.7)	0.002
Alberta	4.1 (2.4–6.3)	9.3 (7.2–11.8)	17.1 (14.9–19.5)	36.4 (34–38.8)	43.8 (41.5–46.1)	50.9 (48.8–53.1)	65.9 (63.8–67.9)	75.2 (73.5–76.9)	82.5 (81−84)	83.7 (82.4–84.9)	80.9 (79.4–82.3)	<0.001
Saskatchewan	0 (0–5.9)	4.3 (1.2–10.8)	16.9 (10.9–24.5)	12.7 (7.3–20.1)	17.3 (12.7–22.6)	34.8 (27.4–42.9)	42.9 (35.3–50.7)	45.3 (39–51.6)	53.5 (46.6–60.4)	69.8 (63.8–75.4)	65.3 (57.6–72.4)	<0.001
Manitoba	2.6 (0.3–9.2)	8 (3.5–15.2)	6.1 (2–13.7)	16.8 (10.7–24.5)	32.9 (26–40.5)	39.8 (33.9–45.9)	38 (32.1–44.1)	54.8 (48.7–60.9)	72.4 (67.6–76.9)	77.2 (72.8–81.3)	70.8 (66.9–74.4)	<0.001
Ontario	2.6 (2.1–3.1)	5.6 (4.9–6.3)	8.1 (7.3–9)	10.3 (9.5–11.2)	20.1 (18.9–21.3)	38.6 (37.3–39.8)	50 (48.8–51.3)	62.2 (61.1–63.2)	71.2 (70.2–72.2)	70.3 (69.5–71.1)	68.6 (67.8–69.4)	<0.001
Quebec	1.5 (0.7–2.7)	7.1 (5.4–9)	5.7 (4.2–7.5)	5.5 (4.2–7.2)	14.6 (12.4–17)	19.6 (17.5–21.8)	18.9 (17–21.1)	13.1 (11.3–15.2)	17.3 (15.1–19.7)	29.1 (26.6–31.7)	19.1 (17.1–21.1)	0.013
Atlantic Canada	1.3 (0.4–2.9)	0.2 (0–1.1)	0 (0–0.8)	1.4 (0.6–2.8)	6.4 (4.3–9.2)	11.5 (8.8–14.6)	8.7 (6.4–11.5)	5.7 (3.7–8.3)	9.1 (6.4–12.5)	6.5 (4.5–9)	15.8 (13.3–18.6)	0.013

All values are displayed as percentages (except p-values). 95% confidence intervals are in parentheses. All p-values were statistically significant after Holm-Bonferroni adjustment (α = 0.05 and n = 8 tests).

**Fig. 2:**
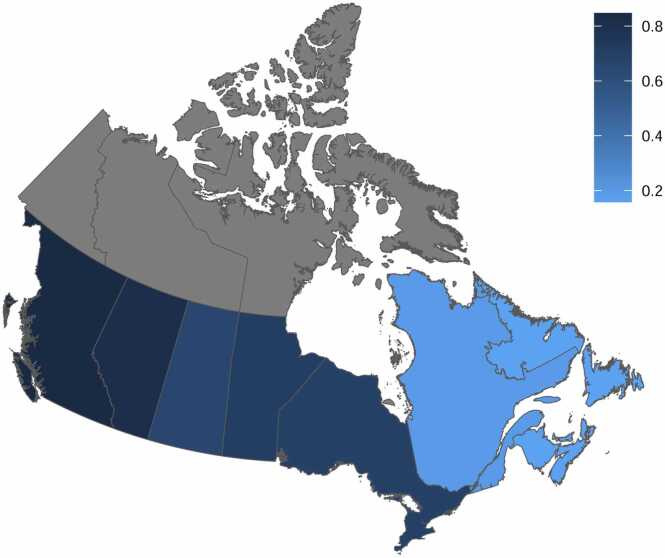
Proportion of opioid-containing samples with fentanyl or a fentanyl analogue, by province/region, 2022. Data from the territories (grey) were not analyzed.

In Quebec, the proportion of fentanyl or a fentanyl analogue within the illicit opioid supply increased from 1.5% (0.7–2.7%) to 19.1% (17.1–21.1%) between 2012 and 2022, while in Atlantic Canada, the proportion increased from 1.3% (0.4–2.9%) to 15.8% (13.3–18.6%). There were statistically significant increases in fentanyl supply in Atlantic Canada and all other provinces, after Holm-Bonferoni adjustment for multiple testing (Table 1). In 2022, there was an east-to-west gradient in the proportion of fentanyl or fentanyl analogues in opioid-containing samples (Fig. 2).

#### 3.1.1 Fentanyl-specific positivity

**Fig. 3:**
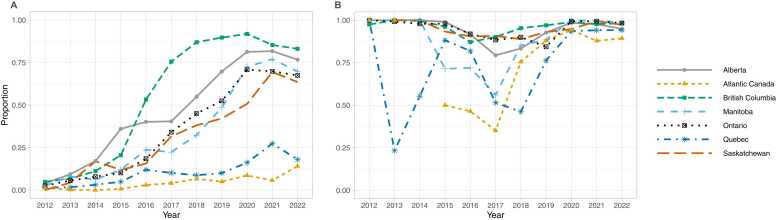
Fentanyl in seized opioids by province/region, 2012–2022. (A) Proportion of illicit opioid-containing samples with fentanyl. (B) Proportion of samples with fentanyl or a fentanyl analogue that contain fentanyl.

Across all provinces, the rate of fentanyl-specific positivity (e.g. presence of fentanyl with or without a fentanyl analogue within the sample) in opioid-containing samples was 2.9% (2.5% - 3.3%) in 2012, which increased to 66.6% (66.0–67.2%) in 2022 (Supplementary Table 1). Trends in fentanyl-specific positivity were broadly similar to trends in positivity for fentanyl or a fentanyl analogue (Fig. 3a). Although fentanyl was present nationally in >95% of samples containing fentanyl or a fentanyl analogue in most years, this was lower in 2016 – 2019 (Fig. 3b). Quebec, Ontario and Atlantic Canada had lower levels of fentanyl-specific positivity in samples containing fentanyl or fentanyl analogue until 2020.

#### 3.1.2 Carfentanil-specific positivity

**Fig. 4:**
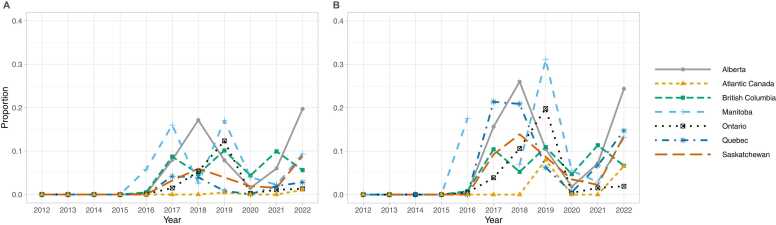
Carfentanil in seized opioids by province/region, 2016–2022. (A) Proportion of illicit opioid-containing samples with carfentanil. (B) Proportion of samples with fentanyl or a fentanyl analogue that contain carfentanil. Carfentanil was first detected by the Health Canada Drug Analysis Service in 2016.

Carfentanil was first detected in drug samples analyzed by the DAS in 2016. Carfentanil was present in 0.3% (0.2–0.4%) of opioid-containing samples in 2016 rising to 10.0% (9.5–10.4%) of opioid-containing samples in 2019 and decreasing to 4.9% (4.6–5.2%) of opioid-containing samples in 2022 (Supplementary Table 2). There were pronounced provincial/regional differences in the proportion of opioid-containing samples containing carfentanil (Fig. 4a).

Carfentanil was present in 0.8% (0.6–1.2%) of samples that contained fentanyl or a fentanyl analogue in 2016 rising to 14.9% (14.2–15.5%) in 2019 and decreasing to 7.2% (6.8–7.6%) in 2022. The proportion of samples with fentanyl or fentanyl analogues that contained carfentanil was variable between provinces / regions (Fig. 4b). The increased national rate of carfentanil positivity in 2019 was driven in part by a single-year increase in the positivity rate in Ontario. As of 2022 in Alberta, 24.4% (22.7–26.1%) of samples containing fentanyl or an analogue contained carfentanil. Carfentanil was present in over 10% of all samples containing fentanyl or an analogue in Saskatchewan, Manitoba and Quebec.

#### 3.1.3 Other analogue-positivity

Fentanyl analogues other than carfentanil were present in under 10% of opioid-containing samples in Canada in all years (Supplementary Table 3). Fentanyl analogues other than carfentanil were present in between 10% and 20% of opioid-containing samples in British Columbia during 2016 – 2018 and in Alberta in 2017 – 2018 and were present in under 10% of samples in all other regions and years (Supplementary Table 3).

**Fig. 5:**
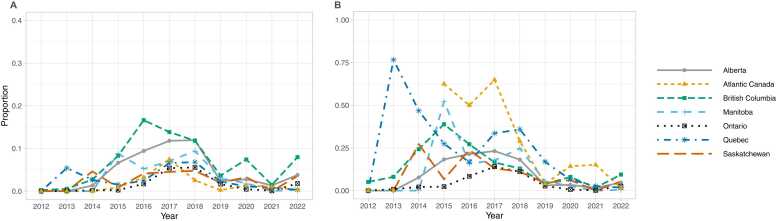
Fentanyl analogues, other than carfentanil, in illicit opioid supply by province/region, 2012–2022. (A) Proportion of illicit opioid-containing samples with fentanyl analogues other than carfentanil. (B) Proportion of samples with fentanyl or a fentanyl analogue containing a fentanyl analogue other than carfentanil.

Across Canada, fentanyl analogues other than carfentanil were present in 22.0% (20.1–24.0%) of samples containing fentanyl or an analogue in 2015, which decreased to 4.5% (4.2–4.9%) by 2022. Similar trends were observed in most regions, though the proportion of fentanyl-analogue positivity varied between regions (Fig. 5). By 2022, the percentage of samples containing a fentanyl analogue was under 10% in all regions.

## 4 Discussion

This study highlights that opioid samples in Canada increasingly contained fentanyl or fentanyl analogues between 2012 and 2022 and that important regional variability existed in these trends. The proportion of opioid samples containing these substances increased first in British Columbia and Alberta, followed shortly by Saskatchewan, Manitoba and Ontario. As of 2022, the proportion of seized opioids positive for fentanyl or a fentanyl analogue followed an east-to-west gradient, with rates lowest in Atlantic Canada and highest in BC. Although over two-thirds of opioid-containing samples nationally in 2022 were positive for fentanyl or fentanyl analogues, the proportions were substantially lower in Quebec and Atlantic Canada.

Carfentanil, a synthetic opioid that is approximately 100 times more potent than fentanyl and 10,000 times more potent than morphine, was first detected in 2016 and was present in 4.9% of opioid-containing samples nationally in 2022 (Delcher, 2020). However, important differences existed between regions. In Alberta, nearly 1 in 5 opioid-containing samples contained carfentanil in 2022, while in Ontario and Atlantic Canada, carfentanil was present in less than 2% of all opioid-containing samples. By 2022, other fentanyl analogues were present in under 4% of opioid-containing samples in all regions of Canada except British Columbia.

Drug testing through using high-performance laboratory evaluation provides important insights into the drug supply for surveillance. Drug checking using test strips, though more cost-effective, can have reduced sensitivity and specificity for detecting compounds. Data from human samples (e.g. urine drug testing or post-mortem serum toxicology) are often unable to distinguish between drugs used over the prior few days prior to testing.

Previous study have found important associations between the presence of fentanyl within the opioid supply and drug-related harms. In Vancouver, Canada the mean fentanyl concentration of samples submitted to drug checking services has been associated with rates of overdose (Kennedy et al., 2023). Similarly, across the US, the proportion of fentanyl within drug seizures by law enforcement agencies have been associated with rates of opioid-involved overdose deaths (Zibbell et al., 2023).

Overdose deaths in Canada increased as fentanyl became more prevalent within the illicit opioid supply. In British Columbia, the rate of drug overdose deaths increased from 11.1 per 100,000 in 2015 to 20.5 per 100,000 in 2016, as the proportion of the illicit opioid supply containing fentanyl increased from 21.3% to 61.2% (British Columbia Coroner Service, n.d). In Ontario, the rate of drug overdose deaths increased from 6.2 per 100,000 in 2016 to 10.4 per 100,000 in 2018, as the proportion of fentanyl and fentanyl analogues substances within the illicit opioid supply increased from 20.1% to 45.0% in 2018 (Public Health Ontario, 2023).

This analysis makes several important findings with implications for clinical care of individuals who use opioids in Canada. First, in provinces west of Quebec, fentanyl was the predominant opioid within samples of seized illicit opioids as of 2022, and treatment strategies for individuals with OUD should respond to this increasingly toxic supply (Bromley et al., 2021, Fiellin, 2022, Kleinman, 2023, Kleinman et al., 2022, Kleinman and Wakeman, 2022, Pardo, 2022, Thakrar and Kleinman, 2022). Second, lower proportions of fentanyl within the illicit opioid suppy in Quebec and Atlantic Canada (below 20% of opioid samples fentanyl-positive in 2022), suggest that these regions may remain susceptible to large increases in opioid-involved overdose deaths if the proportions of fentanyl increase within these regions. Ongoing monitoring and public health efforts are necessary to mitigate against the potential for the rapid increases in overdoses as fentanyl-positivity of opioids increases in these regions. Third, the rate of carfentanil-positivity has had substantial, region-specific annual fluctuations, with high rates of carfentanil-positive sample most recently in Alberta. Fourth, this study highlights how trends at a federal level may obscure important regional differences in drug supply patterns. Analyses of the illicit drug supply in Canada and other large, federated countries, such as the United States, may benefit from stratified analyses at the regional or state level. Fifth, providing data about the proportion of fentanyl within the illicit opioid supply help contextualize the findings of from epidemiologic studies of interventions to assist with opioid use disorder generated from population-based administrative claims data from Canada (Gomes et al., 2022, Pearce et al., 2020). Sixth, this study further highlights the importance of timely release of data about illicit drug trends (including types of synthetic opioids present) to allow tailoring approaches for reducing opioid-related harms to local needs.

This study complements previous work evaluating drug supply at a local level in Canada and in the US. Drug checking services are available in British Columbia and in select urban centres across the rest of the country. These services allow people who use drugs to voluntarily submit drug samples for an analysis of chemical constituents. In British Columbia, drug checking services found that 86% of opioid samples contained fentanyl in 2022, aligning with the findings in this study. This study provides important data from other jurisdictions across Canada and complementary evidence given potential differences between law enforcement drug seizures and drugs being submitted for analysis by people who use drug checking services.

This study has several limitations. First, this study reports on the proportion of fentanyl-positive opioid-containing samples that were submitted to Canada’s federal drug analysis service. Samples are submitted to the drug analysis service voluntarily by law enforcement agencies and do not represent a random sample of either drugs seized by law enforcement agencies or the broader illicit drug supply within Canada. Second, this analysis focuses on fentanyl and its analogues and did not include information about nitazenes or non-opioid toxic contributors to the illicit opioid supply in Canada. Third, this analysis does not account for the concentration of fentanyl, carfentanil, or other analogues within submitted samples, which has been associated with overdose deaths (Kennedy et al., 2023). Fourth, this analysis did not incorporate information about whether fentanyl or its analogues would have been expected within submitted samples, which may affect the potential toxicity to individuals using substances.

## 5 Conclusions

This study characterizes the increasing proportion of opioid samples seized across Canada that contain fentanyl and highlights important regional variability in these trends. The regional variability indicates a need to tailor treatment and public health approaches. In provinces west of Quebec, treatment strategies and policy initiatives for assisting individuals who use opioids must reflect that fentanyl is now present in two-thirds or more of opioid samples. In Quebec and Atlantic Canada, policies should address the both the need to assist individuals exposed to fentanyl and carfentanil through the illicit opioid supply and the prevention of rapid increases in opioid-involved overdose deaths as fentanyl becomes more prominent within the illicit opioid supply.

## Funding sources

Centre for Addiction and Mental Health Discovery Fund

## Author disclosures

This work was funded by the Centre for Addiction and Mental Health Discovery Fund.

## Disclosures of financial or other potential conflicts of interest

None

## Disclaimer

Findings presented here will differ from other data from the Health Canada Drug Analysis Service as these data are presented and analyzed in a different manner. Additional information about the work of the Health Canada Drug Analysis Service can be found here: https://health-infobase.canada.ca/drug-analysis-service/analyzed-drug-report.html.
