# Supplementary material for: Fentanyl, carfentanil and other fentanyl analogues in Canada’s illicit opioid supply: A cross-sectional study
Source: Drug Alcohol Depend Rep. 2024 May 23;12:100240. doi: 10.1016/j.dadr.2024.100240 (PMC11259693; doi:10.1016/j.dadr.2024.100240)
Supplement: Supplementary file 1 — Supplementary material [file mmc1.docx]

**Supplementary Materials.**

Kleinman RA. Fentanyl, carfentanil and other fentanyl analogues in Canada’s illicit opioid supply: A cross-sectional study.

Table Page

Supplementary Table 1: Fentanyl-specific positivity by province/region and year 2

Supplementary Table 2: Carfentanil-positivity by province/region and year 3

Supplementary Table 3: Other fentanyl-analogue positivity by province/region and year 4

**Supplementary Table 1.** Percentage of illicit opioid-containing samples with fentanyl, by province/region and year.

|  | **2012** | **2013** | **2014** | **2015** | **2016** | **2017** | **2018** | **2019** | **2020** | **2021** | **2022** |
| --- | --- | --- | --- | --- | --- | --- | --- | --- | --- | --- | --- |
| Total | 3.0 (2.6-3.4) | 5.4 (4.9-5.9) | 9.1 (8.5-9.7) | 15.8 (15.1-16.5) | 30.4 (29.5-31.3) | 43.7 (42.9-44.5) | 54 (53.2-54.8) | 60.5 (59.8-61.2) | 73.6 (72.9-74.2) | 71.4 (70.9-72) | 66.6 (66-67.2) |
| British Columbia | 4.8 (3.8-6) | 6.7 (5.6-7.9) | 11.2 (9.9-12.6) | 20.5 (19.1-22) | 53.3 (51.5-55.1) | 75.6 (74.3-76.8) | 86.9 (85.9-87.9) | 89.7 (88.8-90.6) | 91.8 (91.1-92.6) | 85.4 (84.4-86.3) | 83.1 (82-84.2) |
| Alberta | 4.1 (2.4-6.3) | 9.3 (7.2-11.8) | 17.1 (14.9-19.5) | 36 (33.6-38.4) | 40.2 (37.9-42.4) | 40.4 (38.3-42.5) | 54.9 (52.8-57) | 69.8 (68-71.5) | 81.3 (79.7-82.8) | 81.7 (80.4-83) | 76.7 (75.1-78.2) |
| Saskatchewan | 0 (0-5.9) | 4.3 (1.2-10.8) | 16.9 (10.9-24.5) | 11.9 (6.6-19.1) | 15.6 (11.3-20.8) | 31.6 (24.4-39.6) | 38.1 (30.7-45.9) | 42.1 (36-48.5) | 50.7 (43.8-57.6) | 69.4 (63.4-75) | 63.5 (55.8-70.8) |
| Manitoba | 2.6 (0.3-9.2) | 8.0 (3.5-15.2) | 6.1 (2-13.7) | 12.0 (6.9-19) | 23.7 (17.6-30.7) | 22.3 (17.5-27.8) | 32.3 (26.7-38.3) | 48.5 (42.4-54.7) | 72.4 (67.6-76.9) | 77 (72.6-81) | 69.8 (65.9-73.5) |
| Ontario | 2.6 (2.1-3.1) | 5.6 (4.9-6.3) | 7.9 (7.1-8.8) | 10.1 (9.3-11) | 18.5 (17.3-19.7) | 34.1 (32.9-35.3) | 45.0 (43.8-46.3) | 52.5 (51.4-53.5) | 70.8 (69.8-71.8) | 69.8 (69-70.7) | 67.4 (66.6-68.3) |
| Quebec | 1.5 (0.7-2.7) | 1.6 (0.9-2.7) | 3.1 (2.1-4.6) | 4.9 (3.6-6.5) | 12 (10-14.2) | 10.1 (8.5-11.8) | 8.7 (7.3-10.3) | 10 (8.4-11.8) | 16.2 (14-18.5) | 27.4 (24.9-29.9) | 18 (16.1-20) |
| Atlantic Canada | 1.3 (0.4-2.9) | 0.2 (0-1.1) | 0 (0-0.8) | 0.7 (0.2-1.8) | 3.0 (1.6-5.1) | 4.0 (2.5-6.2) | 6.6 (4.6-9.1) | 5.0 (3.2-7.5) | 8.6 (6-11.9) | 5.7 (3.8-8.1) | 14.1 (11.7-16.8) |

All values are displayed as percentages.

**Supplementary Table 2.** Percentage of illicit opioid-containing samples with carfentanil, by province/region and year.

|  | **2016** | **2017** | **2018** | **2019** | **2020** | **2021** | **2022** |
| --- | --- | --- | --- | --- | --- | --- | --- |
| Total | 0.3 (0.2-0.4) | 5 (4.7-5.4) | 6.5 (6.1-6.9) | 10 (9.5-10.4) | 1.7 (1.5-1.9) | 3.9 (3.6-4.1) | 4.9 (4.6-5.2) |
| British Columbia | 0.5 (0.3-0.8) | 8.7 (7.9-9.6) | 4.8 (4.2-5.4) | 10.1 (9.3-11) | 4.4 (3.8-5) | 9.9 (9.1-10.8) | 5.6 (5-6.3) |
| Alberta | 0.2 (0-0.5) | 8 (6.9-9.2) | 17.1 (15.6-18.8) | 7.9 (6.9-9) | 1.5 (1.1-2.1) | 6 (5.2-6.9) | 19.7 (18.3-21.2) |
| Saskatchewan | 0 (0-1.5) | 3.2 (1.1-7.4) | 6 (2.9-10.7) | 3.9 (1.9-7.1) | 1.9 (0.5-4.7) | 1.6 (0.4-4) | 8.8 (5-14.1) |
| Manitoba | 5.8 (2.8-10.4) | 16 (11.8-20.9) | 2.6 (1.1-5.3) | 17 (12.8-22.1) | 4 (2.2-6.5) | 2.2 (1-4.2) | 9.3 (7.1-12) |
| Ontario | 0.1 (0-0.2) | 1.5 (1.2-1.8) | 5.3 (4.7-5.9) | 12.3 (11.6-13.1) | 0.3 (0.2-0.4) | 1.1 (0.9-1.3) | 1.3 (1.1-1.5) |
| Quebec | 0 (0-0.4) | 4.2 (3.2-5.4) | 4 (3-5.1) | 0.8 (0.4-1.5) | 0.1 (0-0.5) | 2 (1.3-2.9) | 2.8 (2-3.8) |
| Atlantic Canada | 0 (0-0.8) | 0 (0-0.7) | 0 (0-0.7) | 0.5 (0.1-1.6) | 0 (0-1) | 0 (0-0.7) | 1.0 (0.4-2) |

All values are displayed as percentages. Carfentanil was not detected in any samples prior to 2016.

**Supplementary Table 3.** Percentage of illicit opioid-containing samples with fentanyl analogues (other than carfentanil), by province/region and year.

|  | **2012** | **2013** | **2014** | **2015** | **2016** | **2017** | **2018** | **2019** | **2020** | **2021** | **2022** |
| --- | --- | --- | --- | --- | --- | --- | --- | --- | --- | --- | --- |
| Total | 0.1 (0-0.1) | 0.7 (0.5-0.9) | 1.2 (1-1.5) | 3.6 (3.3-4) | 7.3 (6.8-7.8) | 9 (8.5-9.5) | 8.4 (8-8.9) | 2.3 (2.1-2.5) | 2.9 (2.7-3.2) | 0.6 (0.5-0.8) | 3.1 (2.9-3.3) |
| British Columbia | 0.3 (0.1-0.6) | 0.5 (0.3-1) | 2.7 (2.1-3.5) | 8.3 (7.3-9.4) | 16.6 (15.3-18) | 13.9 (12.8-14.9) | 11.8 (10.9-12.8) | 3.6 (3.1-4.2) | 7.4 (6.7-8.2) | 1.5 (1.2-1.8) | 8 (7.2-8.8) |
| Alberta | 0 (0-0.8) | 0 (0-0.6) | 1.3 (0.7-2.2) | 6.6 (5.5-8) | 9.4 (8.1-10.8) | 11.8 (10.4-13.2) | 12 (10.6-13.4) | 2.3 (1.8-3) | 2.8 (2.2-3.5) | 1.3 (1-1.8) | 3.8 (3.1-4.5) |
| Saskatchewan | 0 (0-5.9) | 0 (0-3.9) | 4.6 (1.7-9.8) | 0.8 (0-4.6) | 4.1 (2-7.4) | 4.5 (1.8-9.1) | 4.8 (2.1-9.2) | 2 (0.6-4.5) | 3.3 (1.3-6.7) | 0 (0-1.4) | 3.5 (1.3-7.5) |
| Manitoba | 0 (0-4.7) | 0 (0-3.6) | 0 (0-4.4) | 8.8 (4.5-15.2) | 5.2 (2.4-9.6) | 7.1 (4.3-10.8) | 9.4 (6.2-13.6) | 3 (1.3-5.8) | 1.9 (0.7-3.8) | 0 (0-0.9) | 0.3 (0-1.2) |
| Ontario | 0 (0-0.1) | 0 (0-0.2) | 0.2 (0.1-0.4) | 0.2 (0.1-0.4) | 1.7 (1.3-2.1) | 5.4 (4.8-6) | 5.5 (5-6.1) | 1.6 (1.4-1.9) | 0.4 (0.3-0.6) | 0.1 (0-0.1) | 1.8 (1.6-2) |
| Quebec | 0 (0-0.5) | 5.4 (4-7.2) | 2.7 (1.7-4) | 1.5 (0.8-2.5) | 2.4 (1.6-3.6) | 6.6 (5.3-8) | 6.8 (5.6-8.2) | 2.2 (1.5-3.2) | 1.1 (0.6-1.9) | 0.8 (0.4-1.4) | 0.3 (0.1-0.8) |
| Atlantic Canada | 0 (0-0.9) | 0 (0-0.7) | 0 (0-0.8) | 0.9 (0.3-2.1) | 3.2 (1.8-5.3) | 7.5 (5.3-10.1) | 2.5 (1.3-4.3) | 0.2 (0-1.3) | 1.3 (0.4-3) | 1 (0.3-2.3) | 0.3 (0-0.9) |

All values are displayed as percentages.
